# A Unilateral, Pruritic Papular Eruption Following Primigravida Pregnancy

**DOI:** 10.7759/cureus.37951

**Published:** 2023-04-21

**Authors:** Morgan A Rousseau, Emelie E Nelson, Rashid M Rashid

**Affiliations:** 1 Dermatology, John P. and Kathrine G. McGovern Medical School at University of Texas Health Science Center at Houston, Houston, USA; 2 Dermatology, Mosaic Dermatology, Houston, USA

**Keywords:** dermatopathology, hyperpigmentation, pregnancy, pruritus, blaschkolinear, lichen planus blaschkoid

## Abstract

Linear lichen planus (LLP), also known as blaschkolinear or blaschkoid lichen planus, is a rare subtype of lichen planus that presents along the lines of Blaschko. While LLP has been associated with vaccinations, neoplasms, medications, and successive pregnancies, we present a case of LLP following a primary pregnancy. A 29-year-old G1P1 female presented to dermatology for the evaluation of an intensely pruritic, whorled rash confined to her left lower leg that appeared shortly after the birth of her first child. A biopsy of the lesion and subsequent histopathology confirmed the diagnosis of LLP. The patient was treated with topical steroids with minimal response to therapy and declined further treatment.

## Introduction

Linear lichen planus (LLP), also known as blaschkolinear or blaschkoid lichen planus, is a rare subtype of lichen planus that presents along blaschkoid lines [1]. The blaschkoid distribution stems from mosaicism during embryonic epidermal cell migration. A loss of heterozygosity during this critical stage of development leads to basal keratinocytes that are inherently susceptible to the CD8+ T-cell autoimmune-mediated lysis, ultimately predisposing the individual to develop the characteristic lesions observed in LLP [1,2]. LLP has been associated with vaccinations, neoplasms, medications, and successive pregnancies [2]. Here, we present a case of biopsy-confirmed primigravida LLP.

## Case presentation

**Figure 1:**
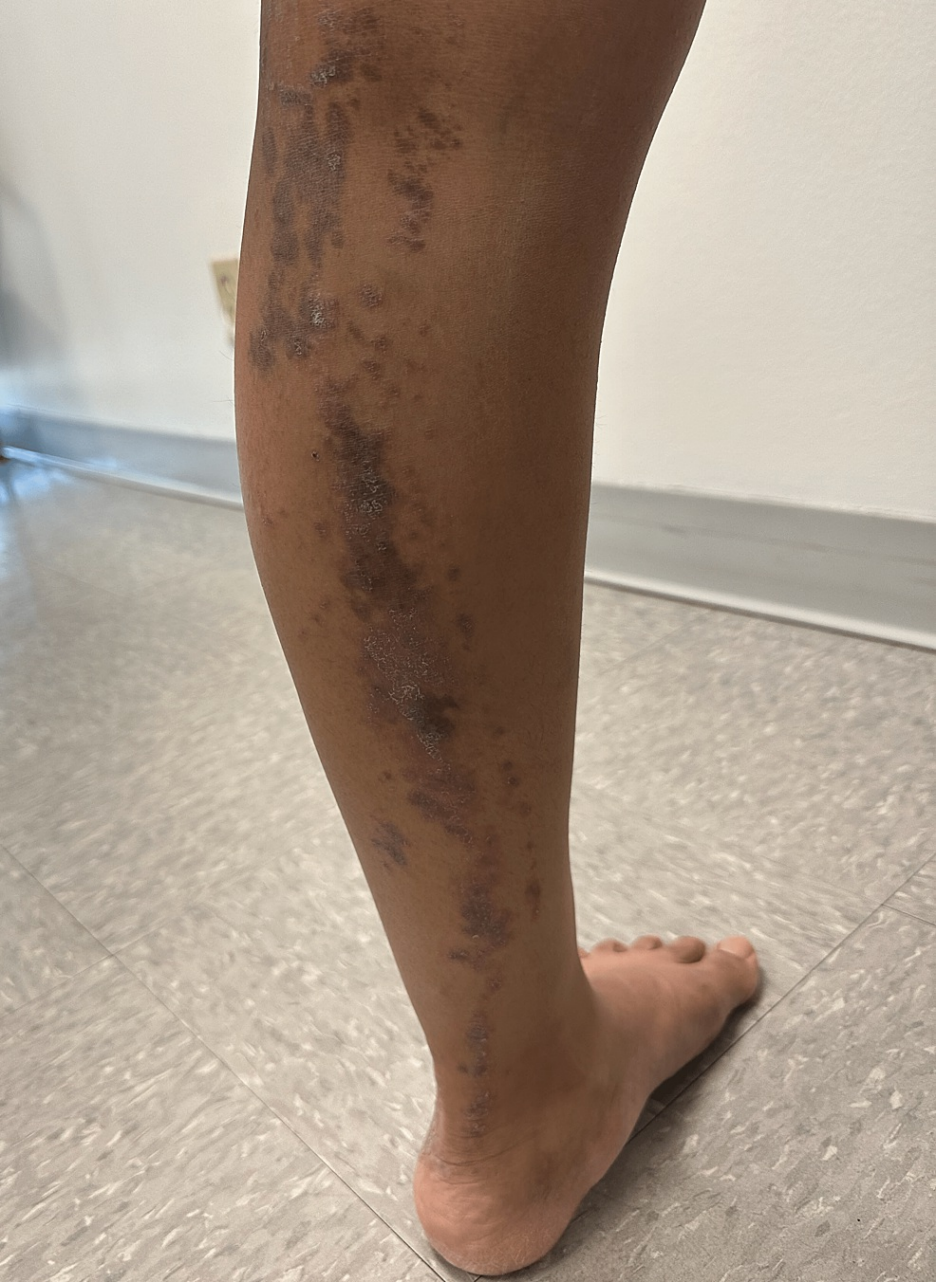
Left lower extremity with excoriated, violaceous, lichenified papules and plaques in a blaschkoid distribution.

**Figure 2:**
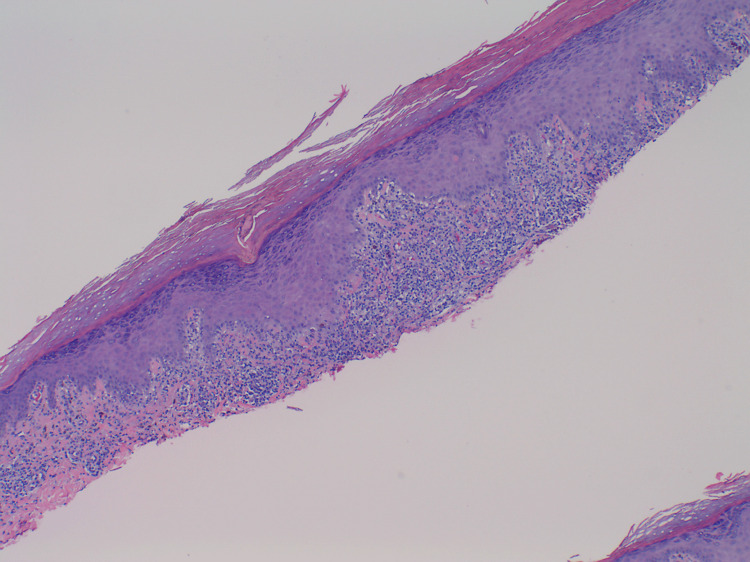
Biopsy stained with hematoxylin and eosin at ×40 magnification demonstrating epidermal and dermal hyperkeratosis and wedge-shaped hypergranulosis, hyperplasia, lymphohistiocytic inflammatory infiltrate with dermoepidermal necrotic keratinocytes at the dermoepidermal junction.

A 29-year-old G1P1 female presented for the evaluation of an intensely pruritic rash localized to her left lower leg that began three months after giving birth to her first child. A physical examination of the patient’s left lower leg revealed excoriated, violaceous, lichenified papules and plaques in a blaschkoid distribution (Figure 1). No other anatomic region demonstrated similar involvement. The patient denied the use of any medications and had not attempted to treat the rash at home. She reported no other significant past dermatologic history, medical history, including no prior history of hepatitis B or hepatitis C, or family history of a similar rash. A biopsy of the lesion revealed epidermal and dermal hyperkeratosis and wedge-shaped hypergranulosis, irregular epidermal hyperplasia, and a lichenoid, lymphohistiocytic inflammatory infiltrate obscuring the dermoepidermal junction. No eosinophils were noted; however, many necrotic keratinocytes were observed at the dermoepidermal junction (Figure 2). Physical examination and histopathology confirmed the diagnosis of LLP. The patient was treated with topical triamcinolone and clobetasol with minimal response to therapy. She was then offered Plaquenil but opted to forgo additional treatment and declined to follow up.

## Discussion

The diagnosis of LLP can be informed by physical examination, dermoscopy, and histologic evaluation. Clinically, LLP lesions resemble the purple, polygonal, violaceous, or erythematous pruritic papules of lichen planus but in a Blaschkoid distribution. Dermoscopy of LLP characteristically reveals white reticular lines, or Wickham striae, and dotted and coiled vessels on a pink background [3]. Histologically, LLP presents with hyperkeratosis without parakeratosis, destruction of the stratum basale, alteration of the rete ridges, and lymphocytic infiltration of the dermoepidermal junction [2].

While LLP has been reported in association with successive pregnancies, this is the first biopsy-confirmed report of LLP presenting postpartum in a primigravida patient [2]. A scoping review of the literature identified two other pregnancy-associated reports of LLP [4,5]. The findings of both cases are summarized in Table 1.

**Table 1:** Linear lichen planus cases that have occurred in association with pregnancy reported to date.

Article	Presentation	Location	Histopathologic description	Management
Krasowska et al., 2001 [4]	Presented postpartum and confirmed after the patient’s third pregnancy	Right upper limb, right chest, and tight epigastrium	Basal vacuolar degeneration	Cimetidine and topical fluticasone propionate
Kumar et al., 2011, [5]	Presented during pregnancy in successive pregnancies	Left hand, left forearm, left shoulder, left flank and abdomen, and left breast	Consistent with lichen planus	Not reported

LLP must be differentiated from other blaschkoid dermatoses, including lichen striatus, inflammatory linear verrucous epidermal nevus (ILVEN), linear psoriasis, and drug-induced blaschkitis. While lichen striatus can morphologically mimic LLP, lichen striatus typically presents in children and is histologically characterized by an epidermal lichenoid infiltrate [2]. ILVEN also presents in infants, progresses slowly, and does not readily respond to treatment [6]. Linear psoriasis is only occasionally pruritic, and histology reveals regular epidermal hyperplasia and Munro microabscesses, characteristic of psoriasis [6]. Finally, the absence of eosinophils on histopathology makes drug-induced blaschkitis less likely.

While LLP typically self-resolves slowly over the course of months, medical management is frequently necessary. Topical glucocorticoids are the first-line therapy, but oral glucocorticoids might be indicated in extensive or refractory cases. Other treatment options include phototherapy and oral antihistamines [1]. Post-inflammatory hyperpigmentation may occur following LLP and can be mitigated with the use of sunscreen and various topical medications.

## Conclusions

While LLP has been reported in association with various vaccinations, neoplasms, medications, and successive pregnancies, our case represents the first biopsy-confirmed case of primigravida LLP eruption presenting after delivery. Because LLP may present similarly to other blaschkoid dermatoses, a biopsy often proves useful in confirming the diagnosis and informing the treatment plan. It is important that dermatologists recognize this condition and treat it appropriately as early intervention can greatly improve patient morbidity.
